# COVID-19 Recommendations for Assisted Living: Implications for the Future

**DOI:** 10.1093/geroni/igab046.217

**Published:** 2021-12-17

**Authors:** Andrew Vipperman, Sheryl Zimmerman, Philip Sloane

**Affiliations:** 1 University of Virginia, Charlottesville, Virginia, United States; 2 Cecil G. Sheps Center for Health Services Research, Chapel Hill, North Carolina, United States; 3 UNC Medical School, Sheps Center, Chapel Hill, North Carolina, United States

## Abstract

Similar to nursing homes, COVID-19 has challenged assisted living (AL), given its congregate nature and vulnerable residents. However, COVID-19 recommendations have not consistently recognized differences between nursing homes and AL, and in so doing present implications for the future of AL. This project examined COVID-19 recommendations from six key organizations and compared them across nursing homes and AL. Differences include recommending more flexible visitation and group activities for AL, while similarities suggest that AL may best integrate health care into offered services (e.g., work with consulting clinicians who know residents and the AL community). Primary points to be discussed are that COVID-19 may accelerate the closer coordination of social work and medical care into AL, because recommendations suggest AL would benefit from the services and expertise of nurses, social workers, and physicians. There seems to be an unmet need to mitigate loneliness in AL, which warrants specific attention moving forward.

